# Single-Cell RNA-Seq of Bone Marrow Cells in Aplastic Anemia

**DOI:** 10.3389/fgene.2021.745483

**Published:** 2022-01-03

**Authors:** Hu Tonglin, Zhao Yanna, Yu Xiaoling, Gao Ruilan, Yin Liming

**Affiliations:** ^1^ Hematology, The First Affiliated Hospital of Zhejiang Chinese Medical University, Hangzhou, China; ^2^ Institute of Hematology, the First Affiliated Hospital of Zhejiang Chinese Medical University, Hangzhou, China

**Keywords:** aplastic anemia, bone marrow, stem cell, BCR, single-cell RNA-seq

## Abstract

Aplastic anemia (AA) is an autoimmune disease characterized by peripheral blood pancytopenia and bone marrow failure. Recently, a research study verified bone marrow failure of AA patients resulting from hematopoietic stem and progenitor cell (HSPC) attack by active T cells. Nonetheless, whether B cells, as one of the important immune cells, destruct the hematopoiesis is still unclear. Here, a large-scale single-cell transcriptomic sequencing of 20,000 bone marrow cells from AA patients and healthy donors was performed. A total of 17 clusters and differentially expressed genes were identified in each cluster relative to other clusters, which were considered potential marker genes in each cluster. The top differentially expressed genes in HSPCs (S100A8, RETN, and TNFAIP3), monocytes (CXCL8, JUN, and IL1B), and neutrophils and granulocytes (CXCL8, NFKBIA, and MT-CYB) were related to immune and inflammatory injury. Then, the B-cell receptor (BCR) diversities and pairing frequencies of V and J genes were analyzed. The highest pairing frequencies in AA patients were IGHV3-20-IGKJ2, IGHV3-20-IGKJ4, and IGHV3-20-IGHLJ2. Meanwhile, there were 3 V genes, including IGHV3-7, IGHV3-33, and IGLV2-11, with elevated expression in B cells from AA patients. Cell type–specific ligand–receptor was further identified in B-cell interaction with hematopoietic cells in the bone marrow. The changed ligand–receptor pairs involved antigen presentation, inflammation, apoptosis, and proliferation of B cells. These data showed the transcriptomic landscape of hematopoiesis in AA at single-cell resolution, providing new insights into hematopoiesis failure related with aberrance of B cells, and provide available targets of treatment for AA.

## Introduction

Aplastic anemia (AA) is an autoimmune disease characterized by peripheral blood pancytopenia and bone marrow failure (Young, 2018; Liu et al., 2019). It may occur at any age; however, young individuals (10–25 years) and the elderly (>60 years) are the most susceptible. No significant differences in gender have been reported. Although the clinical symptoms of AA can be improved by bone marrow transplantation and/or immunosuppressive therapy (Bacigalupo, 2017), the lack of suitable donors and the side effects of immunosuppressant therapy remain problematic (Pierri and Dufour, 2019). Currently, no effective clinical treatments for AA are available.

In AA patients, hematopoietic stem and progenitor cells (HSPCs) show decreased number as well as reduced proliferation and differentiation, and immune cells have abnormal activation. However, it is unclear whether the molecular changes of mRNAs in HSPCs and HPCs due to transcriptome differences among cells are completely masked when population-level RNA-seq is performed. Therefore, it is urgent to identify new marker genes as therapeutic targets.

Most AA cases show effective response to immunosuppressive agents, while some aplastic patients respond well to rituximab treatment, indicating that activated T cells and abnormal B cells destroy hematopoietic cells in AA patients (Bacigalupo, 2017; Zhu et al., 2021). Further studies showed a negative correlation between the amount of regulatory T cells and CD20 + B cells in aplastic patients, suggesting that the inhibitory effect of regulatory T cells on B cells is weakened in aplastic patients (Huuhtanen et al., 2019). Therefore, it is unclear whether clonal expansion of B cells is associated with aplastic B-cell abnormalities.

Interestingly, single-cell RNA-seq (scRNA-seq) is considered a powerful tool for comprehensively dissecting cellular heterogeneity, and these advances have enabled the transcriptomes of tens of thousands of cells to be assayed at single-cell resolution in a single experiment. Substantial data have been obtained by scRNA-seq of T cells and HSPCs in AA patients (Firat et al., 1998; Butler et al., 2018). It is thus of great interest to cease this unprecedented opportunity to dissect the cellular heterogeneity of bone marrow cells from AA patients by large-scale single-cell transcriptomic profiling.

Here, a large-scale single-cell transcriptomic sequencing of 20,000 bone marrow cells from AA patients and healthy donors was performed. A high-quality dataset was provided, which would be a valuable resource for dissecting the intrapopulation heterogeneity as well as interrogating lineage priming patterns for any lineages at single-cell resolution. Meanwhile, the BCR diversities, pairing frequencies of the V and J genes, and ligand–receptor pairs in B-cell interaction with hematopoietic cells in the bone marrow were analyzed.

## Methods

### Cell Preparation

BM samples were collected from two adult healthy donors and two AA patients at the First Affiliated Hospital of Zhejiang Chinese University. All participants provided written informed consent before enrollment in this study. Biospecimen collection protocols complied with local guidelines and were approved by the Ethics Committee of the First Affiliated Hospital of Zhejiang Chinese University. The isolation procedure was described previously (Macosko et al., 2015). Mononuclear cells (MNCs) were isolated using Ficoll–Hypaque gradient separation (Tianjin Hao Yang Biological Products Technology, China). CD34 ^+^ cells were purified from MNCs with the human anti-CD34 MicroBeads Isolation Kit (Miltenyi Biotec) according to the manufacturer’s specifications, and CD34 ^+^ cells were obtained. Then, MNCs mixed with CD34 ^+^ cells at a 4:1 ratio were analyzed by 10× Genomics.

### Single-Cell RNA-Seq Data Preprocessing

The Cell Ranger software pipeline (version 2.2.0) provided by 10× Genomics was used to demultiplex cellular barcodes, map reads to the genome and the transcriptome using the STAR aligner, and down-sample reads as required to generate normalized aggregate data across samples, producing a matrix of gene counts *versus* cells. The unique molecular identifier (UMI) count matrix was processed using the R package Seurat (Mabbott et al., 2013) (version 2.3.4). To remove low-quality cells and likely multiplet captures, which is a major concern in microdroplet-based experiments, a criterion to filter out cells with UMI/gene numbers out of the limit of mean value ± two-fold of standard deviations was applied, assuming a Gaussian distribution of each cell’s UMI/gene numbers. Following visual inspection of the distribution of cells by the fraction of mitochondrial genes expressed, low-quality cells in which >10% of the counts belonged to mitochondrial genes were further discarded. After applying these QC criteria, 20,419 single cells and 33,538 genes in total remained, and were included in downstream analyses. Library size normalization was performed with Seurat on the filtered matrix to obtain the normalized count.

Top variable genes across single cells were identified by the method described by Macosko et al. (Li et al., 2017). Briefly, average expression and dispersion were calculated for each gene, and all genes were subsequently placed into 17 bins based on their expression. Principal component analysis (PCA) was performed to reduce the dimensionality of the log-transformed gene-barcode matrices of top variable genes. The cells were clustered based on the graph-based clustering approach and visualized in two dimensions using tSNE. The likelihood ratio test that simultaneously tests for changes in mean expression and percentage of expressed cells was used to identify significantly differentially expressed genes among clusters. The R package SingleR, a novel computational method for unbiased cell type recognition of scRNA-seq, with two reference transcriptomic datasets “blueprint_encode” (da Silva et al., 2020), was utilized to infer the cell of origin of each of the single cells independently and to identify cell types. RNA-seq data are deposited in the Gene Expression Omnibus database under the accession number GSE145669.

## Results

### Study Participants

Aplastic anemia patients 1 (AA1) and 2 (AA2) were diagnosed with serious aplastic anemia (SAA). Absolute neutrophil count, platelets, and hemoglobin concentration were decreased in both patients. Their clinical characteristics are summarized in Table 1, Supplemental Figure 1, and Supplementary Table S1.

**TABLE 1:** Peripheral blood counts and bone marrow features in patients with aplastic anemia and healthy donors.

Case	Gender	Age (years)	WBC	Neutrophils	Platelets	Hb (g/L)	M/E ratio	Granulocytes (%)	Erythroblasts (%)	Megakaryocytes (%)	Lymphocytes (%)
			10^9^/L	10^9^/L	10^9^/L						
Normal-1	Female	37	6.7	4.4	195	129	2.5	55.0	22.0	More	16.0
Normal-2	Female	47	6.5	4	266	128	3.18	62.0	19.5	More	14.0
AA1	Female	56	1.3	0.5	23	69	0.76	36.0	47.0	Rare	14.5
AA2	Male	21	1.5	0.4	9	67	0.2	6.0	30.0	Rare	58.5

The features of BM cytology for these patients are described in Table 1. AA patients presented abnormalities in hematopoietic cells and non-hematopoietic cells. The percentage of hematopoietic cells was decreased in the bone marrow of AA patients, while that of non-hematopoietic cells was increased.

### Single-Cell RNA-Seq Identifies Multiple Cell Populations in the Bone Marrow

The 10× Genomics Chromium platform was employed to construct single-cell RNA-seq libraries of four bone marrow samples, including two each from healthy donors and AA cases. Saturation curve analysis indicated that the sequencing depth was almost sufficient for gene detection in each sample, and the median numbers of genes detected per cell were comparable among the four samples (Supplementary Figure S2A). The distribution of the three data quality metrics, that is, the proportion of UMI counts for mitochondrial genes, the number of genes detected, and the sum of UMI counts in each cell, fitted a generalized liner model by filtering delocalized cells among the four samples. To exclude potential low-quality data that may result from broken cells, multiples, or other technical issues, stringent thresholds were set for the three data quality metrics, and high-quality data for 20,000 cells were finally retained (Supplementary Figure S2B and Supplementary Table S2). Taken together, a high-quality large-scale single-cell transcriptomic dataset of cells from bone marrow specimens was generated.

**FIGURE 1:**
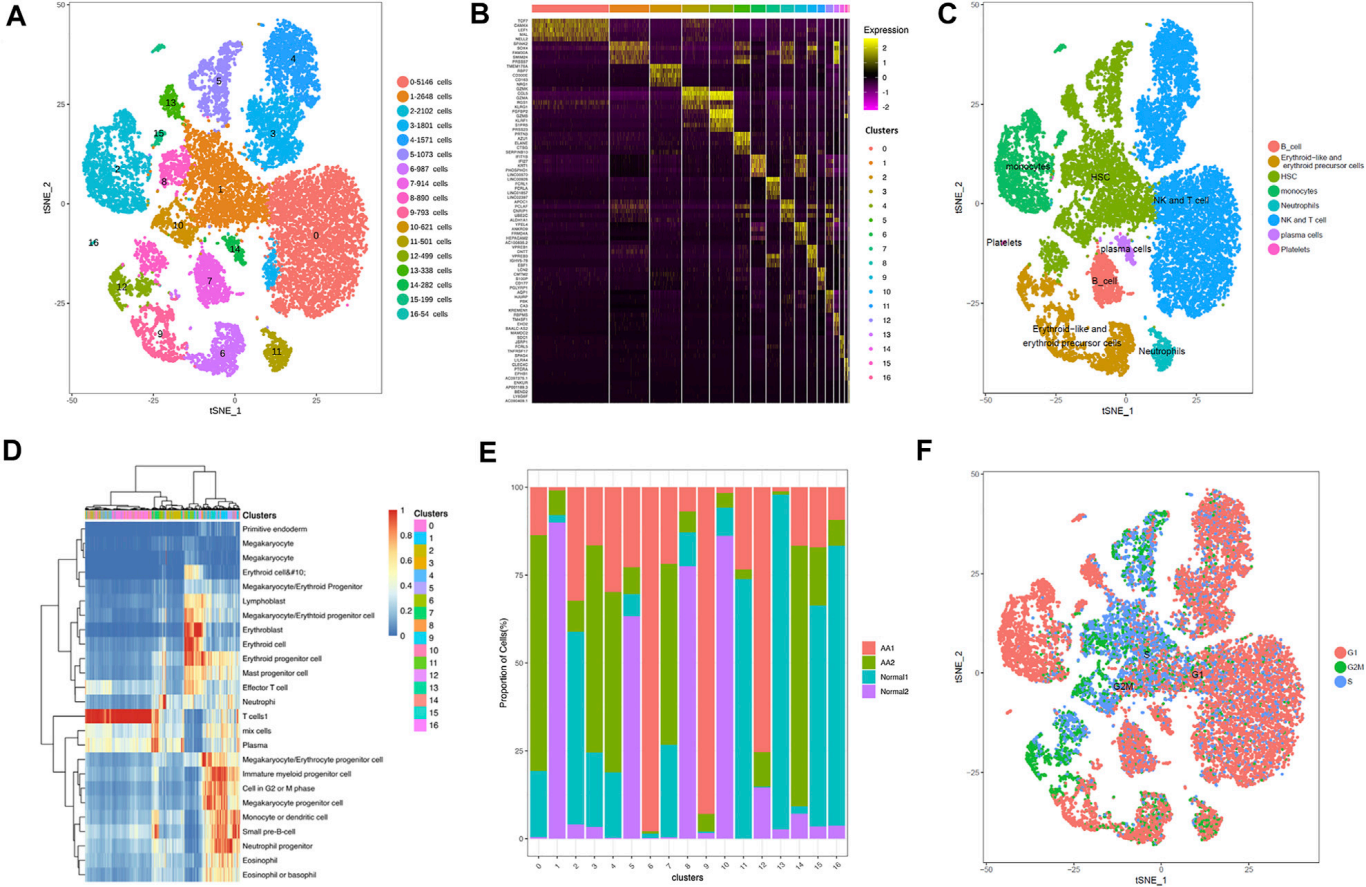
Overview of cell heterogeneity in samples from AA patients and healthy donors. Totally 17 cell clusters were identified by scRNA-seq. **(A)** Totally 17 cell clusters were identified by heat-distributed stochastic neighbor embedding (tSNE). **(B)** Marker genes of each cell cluster. **(C)** Cell type identification and distribution in each cell cluster. **(D)** Heatmap depicting representative differentially expressed genes from each cell cluster. **(E)** Proportions of cell clusters in each sample. **(F)** Cycle of each cell cluster.

The single-cell sequencing data matrix contained tens of thousands of genes, which made cluster analysis difficult. In order to obtain better clustering results, the t-distributed stochastic neighbor embedding (tSNE) dimension reduction algorithm was used to reduce the dimension and to cluster the obtained single-cell UMI quantitative matrix; 2 (2D) or 3 (3D) dimensions were taken for clustering. The tSNE program was used to display the results of single-cell RNA-seq data. The 17 clusters were identified, and their distribution in the AA and healthy donor groups was analyzed (Figure 1A). Through differential gene analysis, genes differentially expressed in each cell group to other cell groups were identified, constituting potential marker genes in each cell group. SingleR was used to determine the correlation between the expression profile of the identified cells and the reference dataset; the cell type with the highest correlation in the reference dataset was assigned to the identified cells. The visualization diagrams of marker genes in various cell clusters in tSNE are shown in Figure 1B. Based on the gene expression pattern of each cluster, HSPCs, monocytes, neutrophils, erythrocytes, T cells, B cells, and plasma cells were identified (Figure 1C). Figure 1D shows the heatmap for representative differentially expressed genes in each cluster. The changes in the proportion of each cluster are shown in Figure 1E. Interestingly, HSPCs were mainly expressed in the G2/S phase of the cell cycle, and immune cells were mainly expressed in the G1 phase (Figure 1F).

### HSPCs

The tSNE program was used to display the results of single-cell RNA-seq data. The tSNE method gives a sensitive separation of closely related groups of objects. There were common genes in Clusters 1, 5, 8, 10, and 13, including SPINK2, SOX4, FAM30A, CDK6, AC084033.3, STMN1, SMIM24, PRSS57, MEF2C, and IGLL1. The top 10 marker genes of Clusters 1, 5, 8, 10, and 13 are shown in Supplementary Table S3. Compared with healthy donors, the top differentially expressed genes in AA patients were DEFA4, DEFA3, LYZ, IGKV1-5, IGKV3-20, S100A8, RETN, S100A9, IL7R, TNFAIP3, CXCR4, SOX4, RPS4X, STMN1, IGLL1, and SPINK2. With distinct gene expression patterns and lineage-specific differentiated transcription factors, HSPCs were segregated into a mixed population of hematopoietic stem cells and multipotent progenitors (HSC/MPPs, Cluster 1), granulocyte and monocyte progenitors (GMPs, Cluster 5), megakaryocyte and erythroid progenitors (MEPs, Cluster 8), pre-B cells (Cluster 10), and T lymphoid progenitors (TLPs, Cluster 13).

In KEGG pathway enrichment analysis, upregulated genes were involved in many signaling pathways, including NF-kappa B signaling, T-cell receptor signaling, Th1 and Th2 cell differentiation, Th17 cell differentiation, natural killer cell–mediated cytotoxicity, and oxidative phosphorylation (Supplementary Figure S3). Therefore, HSPCs tended to differentiate into lymphocytes in AA, which may contribute to hematopoietic repression in AA.

### B Cells

**FIGURE 2:**
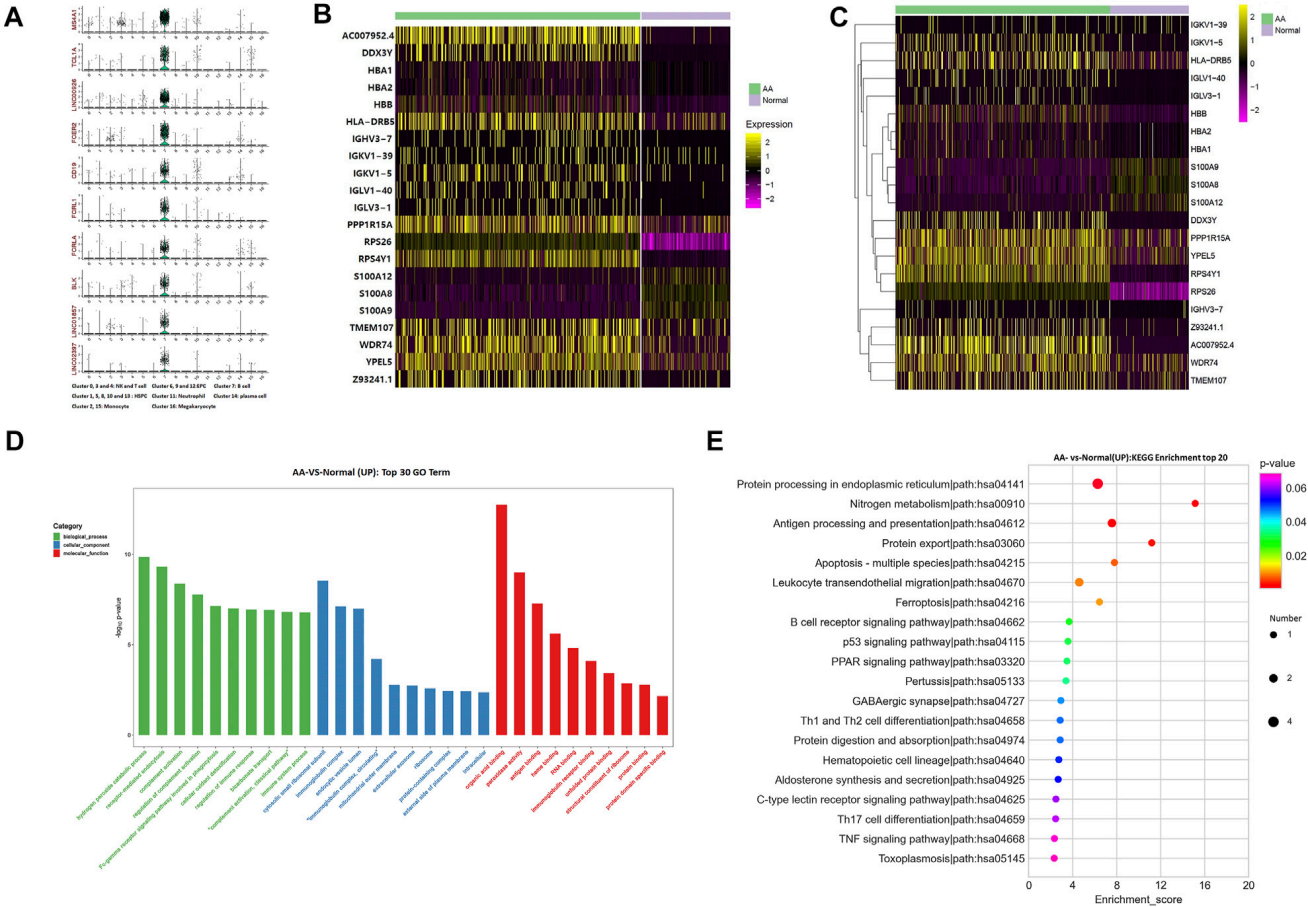
Alterations of B-cell genes in samples from AA patients. **(A)** Violin plot of the top 10 marker genes in B cells. **(B)** Heatmap of differentially expressed genes in B cells. **(C)** Gene clustering of differentially expressed genes in B cells. **(D)** GO analysis of differentially expressed genes in B cells. **(E)** KEGG pathways of differentially expressed genes in B cells.

The top 10 marker genes of Cluster 7 were MS4A1, TCL1A, LINC00926, FCER2, CD19, FCRL1, FCRLA, BLK, LINC01857, and LINC02397 (Figure 2A). CD19 is a known marker for B cells (Nielsen et al., 2020). Therefore, it was confirmed that Cluster 7 cells were B cells. Compared with healthy donors, the top differentially expressed genes in AA patients were involved with protein translation (RPS26 and RPS4Y1), immunoglobulin production (HLA-DRB5, IGHV3-7, IGKV1-39, IGKV1-5, IGLV1-40, and IGLV3-1), IL-17 activation genes (S100A8, S100A9, and S100A12), and cell differentiation (TMEM107) (Figures 2B,C). In the KEGG pathway enrichment analysis, the differentially expressed genes were involved in many signaling pathways, including P53 signaling pathway, Th1 and Th2 cell differentiation pathways, B-cell receptor signaling pathway, and IL-17 signaling pathway, which display a cell-specific expression profile (Figures 2D,E). Therefore, gene alterations in B cells participated in AA pathogenesis.

### BCR Diversities and Pairing Frequencies of V and J Genes

**FIGURE 3:**
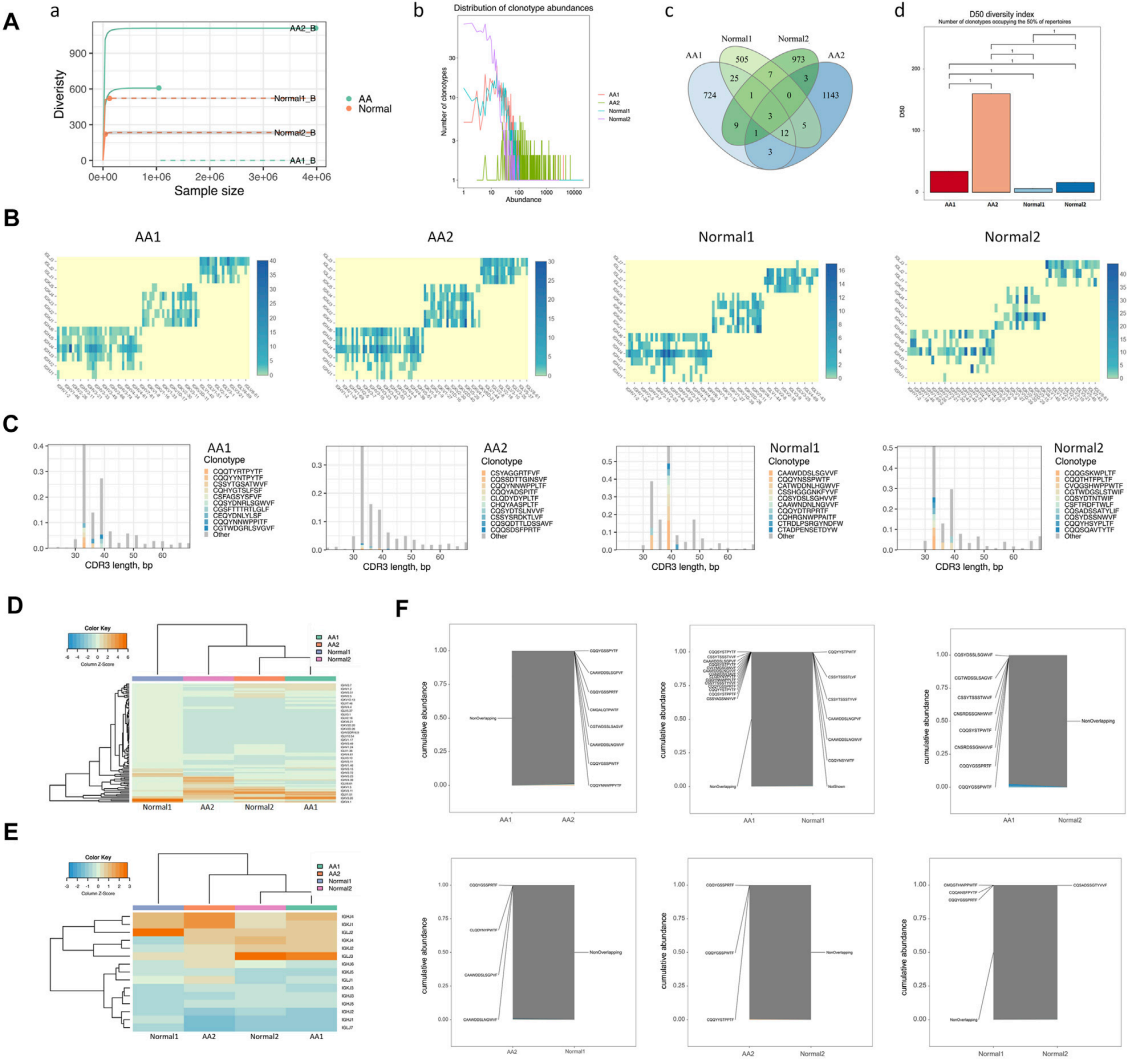
BCR diversities and pairing frequencies of V and J genes. **(A)** BCR diversities, including sample dilution curve (a), rank abundance curve of BCRs (b), venn diagram of BCRs (c), and the D50 index of BCR diversity (d). **(B)** Heatmap depicting V–J gene pair use frequency **(C)** Distribution map of V gene composition with the length distribution of CDR3 amino acid sequences. **(D)** Heatmap of the V gene. **(E)** Heatmap of the J gene. **(F)** Coincidence comparison of immune group libraries.

BCR diversity was analyzed in healthy donors and AA patients by rarefaction curve, rank abundance curve, venn diagram, and D50 index analyses (Figure 3A). The number of BCR collotypes in the AA1 and AA2 samples was 778 and 1,170, respectively. The number of collotypes in both AA1 and AA2 samples was 19 (Figures 3A–C). The D50 value was also higher in AA patients than in the Normal group (Figures 3A–D). Therefore, BCR diversity in AA patients was significantly higher than in healthy donors.

The frequency of V–J gene pairing was higher in the AA group than in the Normal group (Supplemental Table 4 and Figure 3B). The top pairing frequencies in AA patients were IGHV3-20-IGKJ2, IGHV3-20-IGKJ4, and IGHV3-20-IGHLJ2 (Supplemental Figure 4A). For CDR3 amino acid sequence lengths under 30–40 bp, the ratio of the top 10 CDR3 amino acid sequences was lower in the AA group than in the Normal group. For those >40 bp of CDR3 amino acid sequence lengths, the ratios of the top 10 CDR3 amino acid sequences were similar in the AA and Normal groups (Figure 3C). The distribution map of V gene composition with the length distribution of CDR3 amino acid sequences is shown in Supplementary Figure S4B. The 12 high-abundance V genes in the AA group were mainly distributed in 30–40 bp CDR3 amino acids. Meanwhile, the 12 high-abundance V genes in the Normal group were mainly distributed in 30–60 bp CDR3 amino acid sequences. It was speculated that higher pairing frequencies and CDR3 amino acid sequence changes participated in AA pathogenesis.

To assess the unique changes and preferred BCR genes in AA, the use of VDJ genes between AA patients and healthy donors was compared. An overrepresentation of the IGHV3 and IGHV2 families was observed, especially IGHV3-7, IGHV3-33, and IGLV2-11, which were almost not expressed in healthy donors (Figure 3D). In contrast, IGLV1-44 was almost not expressed in AA samples (Figure 3E). The expression pattern of the V gene was incompletely similar in the AA group *versus* that in the healthy donors (Figure 3F). The abovementioned genes may serve as markers for AA diagnosis.

An increase of clonotypes in AA patients together with a skewed use of the IGHV gene suggested that abnormal expression and clonotypes in VDJ genes might contribute to AA pathogenesis. Notably, the abnormal use of dominated IGV genes in AA patients, especially IGHV3-7, IGHV3-20, and IGHV3-33, provided a target for the rational design of monoclonal antibodies.

### Plasma Cells

**FIGURE 4:**
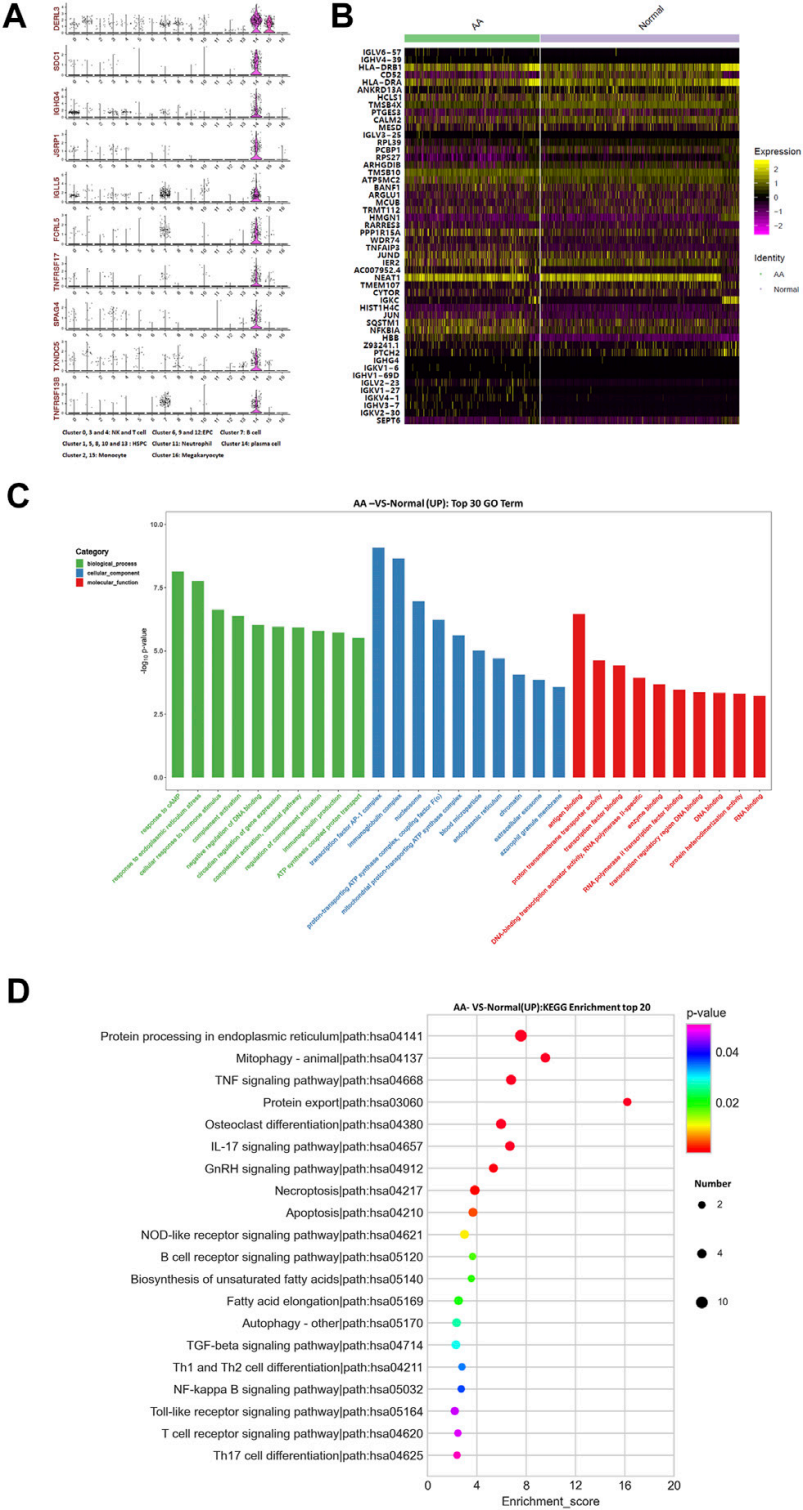
Gene alterations in plasma cells from AA patients. **(A)** Violin plot of the top 10 marker genes in monocytes. **(B)** Heatmap of differentially expressed genes in monocytes. **(C)** GO analysis of upregulated genes in monocytes. **(D)** KEGG pathway of upregulated genes in monocytes.

The top 10 marker genes of Cluster 14 were DERL3, SDC1, IGHG4, JSRP1, IGLL5, FCRL5, TNFRSF17, SPAG4, TXNDC5, and TNFRSF13B (Figure 4A). It was reported that DERL3, SDC1, IGLL5, and FCRL5 are markers of plasma cells (Holm and Hansen, 2020). SDC1 was only expressed in Cluster 14. Therefore, it was confirmed that Cluster 14 cells were plasma cells. Compared with healthy donors, the top differentially expressed genes in AA patients were IGHV3-7, IGHV4-39, IGLV3-25, IGLV6-57, IGKV4-1, IGKV1-27, IGKV2-30, IGLV2-23, IGKV1-6, IGHV1-69D, IGHG4, HLA-DRB1, CD52, HLA-DRA, ANKRD13A, HCLS1, TMSB4X, PTGES3, CALM2, MESD, RPL39, PCBP1, RPS27, ARHGDIB, TMSB10, ATP5MC2, BANF1, ARGLU1, MCUB, TRMT112, HMGN1, RARRES3, PPP1R15A, WDR74, TNFAIP3, JUND, IER2, NEAT1,TMEM107, CYTOR, IGKC, HIST1H4C, JUN, SQSTM1, NFKBIA, PTCH2, and SEPT6 (Figures 4B,C). In the KEGG pathway enrichment analysis, differentially expressed genes were involved in many signaling pathways, including apoptosis, necroptosis, IL-17 signaling pathway, osteoclast differentiation, TNF signaling pathway, mitophagy, and ribosome and oxidative phosphorylation. Importantly, NFKBIA and IL-17 participate in the positive feedback regulation mechanism of monocyte inflammation. Therefore, gene alterations in plasma cells participated in Th17 cell differentiation in AA patients.

### Monocytes

**FIGURE 5:**
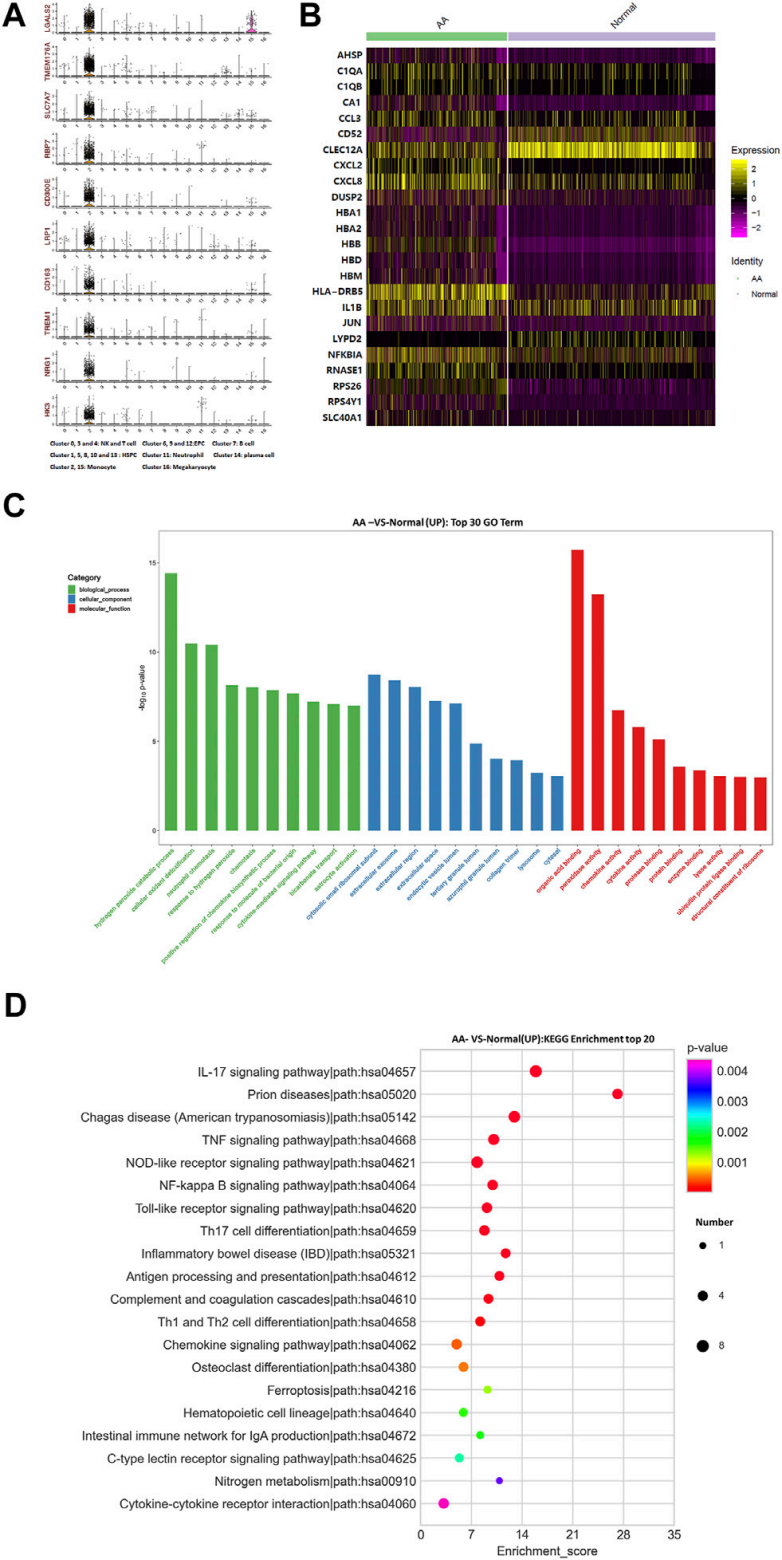
Gene alterations in monocytes from AA patients. **(A)** Violin plot of the top 10 marker genes in monocytes. **(B)** Heatmap of differentially expressed genes in monocytes. **(C)** GO analysis of upregulated genes in monocytes. **(D)** KEGG pathway of upregulated genes in monocytes.

The top 10 marker genes of Clusters 2 and 15 were LGALS2, TMEM176A, SLC7A7, RBP7, CD300E, LRP1, CD163, TREM1, NRG1, and HK3 (Figure 5A). Meanwhile, CD14 is a recognized marker of monocytes and is only expressed in Cluster 2 (Bacigalupo et al., 1980). Therefore, it was confirmed that Cluster 2 cells were monocytes. Compared with healthy donors, the top differentially expressed genes in AA patients were AHSP, C1QA, C1QB, CA1, CCL3, CD52, CLEC12A, CXCL2, CXCL8, DUSP2, IL1B, JUN, LYPD2, NFKBIA, RNASE1, RPS26, RPS4Y1, SLC40A1, *etc* (Figure 5B). The KEGG pathway enrichment analysis indicated that differentially expressed genes were involved in many signaling pathways, including IL-17 signaling pathway, TNF signaling pathway, NF-kappa B signaling pathway, Th17 cell differentiation, and NOD-like receptor signaling pathway. Importantly, NFKBIA and IL-17 participate in the positive feedback regulation mechanism of monocyte inflammation. Therefore, monocytes were in a state of inflammatory activation and participated in Th17 cell differentiation in AA patients.

### Neutrophil Granulocytes

**FIGURE 6:**
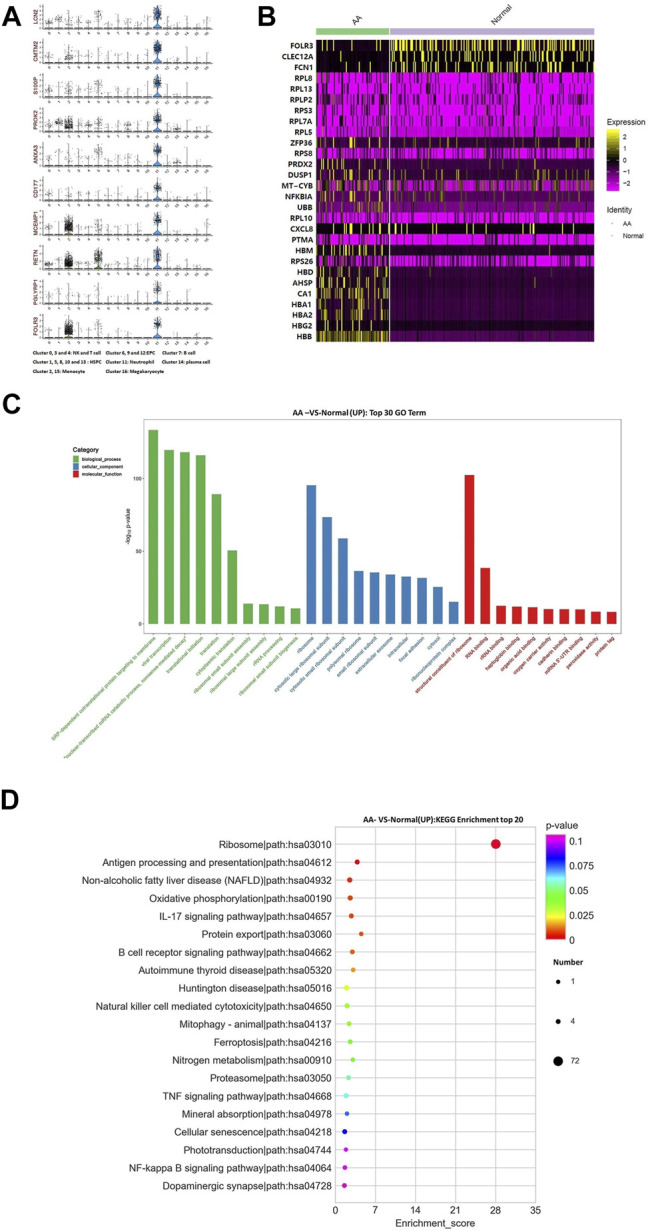
Gene alterations in neutrophil granulocytes from AA patients. **(A)** Violin plot of the top 10 marker genes in neutrophil granulocytes. **(B)** Heatmap of differentially expressed genes in neutrophil granulocytes. **(C)** GO analysis of upregulated genes in neutrophil granulocytes. **(D)** KEGG pathways of upregulated genes in neutrophil granulocytes.

The top 10 marker genes of Cluster 11 were LCN2, CMTM2, S100P, PROK2, ANXA3, CD177, MCEMP1, RETN, PGLYRP1, and FOLR3 (Figure 6A). FOLR3 is specific for neutrophil granulocytes, which function as antimicrobial and antitumor cells. The expression of FOLR3 in AA patients was lower than that of Normal donors. Therefore, it was confirmed that Cluster 11 cells were neutrophil granulocytes. Compared with healthy donors, the top differentially expressed genes in AA patients were CA1, AHSP, RPS26, PTMA, CXCL8, RPL10, UBB, NFKBIA, MT-CYB, DUSP1, PRDX2, RPS8, ZFP36, RPL5, RPL7A, RPS3, RPLP2, RPL13, RPL8, FCN1, FOLR3, CLEC12A, *etc* (Figure 6B). In the KEGG pathway enrichment analysis, these differentially expressed genes were involved in many signaling pathways, including ribosome, antigen procession and presentation, oxidative phosphorylation, B-cell receptor signaling pathway, and IL-17 signaling pathway. Interestingly, genes associated with ribosomal protein (RP) were upregulated, including RPL8, RPL13, RPLP2, RPS3, RPL7A, RPL5, RPS8, and RPS26. It was speculated that the upregulation of RP genes was associated with abnormal neutrophil granulocytes in AA patients.

### Erythroblasts

There were common genes in Clusters 6, 9, and 12, including SELENBP1, GMPR, IFI27, DMTN, KRT1, PHOSPHO1, AC130456.3, and LINC00570. The top 10 marker genes of Cluster 6 were SELENBP, IFIT1B, GMPR, IFI27, DMTN, KRT1, PHOSPHO1, AC130456.3, PDZK1IP1, TMCC2, and LINC00570. The top 10 marker genes of Cluster 9 were SPTA1, SOX6, YPEL4, TSPO2, ANKRD9, FHDC1, FRMD4A, HEPACAM2, AC100835.2, and SLFN14. The top 10 marker genes of Cluster 12 were AQP1, HJURP, A4GALT, TMEM233, PBK, SKA1, CA3, CCDC68, SLC25A21, and KREMEN1. Meanwhile, erythrocyte-specific transcription factors, including GATA-1, GATA-2, and KLF1, were expressed in Clusters 6, 9, and 12. Though the cells belonged to erythrocytes in Clusters 6, 9, and 12, it was speculated that they were in a different cell phase.

### Megakaryocytes

The top 10 marker genes of Cluster 16 were PF4V1, AC147651.1, AP001189.1, ENKUR, AP001189.3, BEND2, LY6G6F, AC090409.1, C15orf54, and TRAPPC3L. These are markers associated with megakaryocytes. The genes associated with megakaryocyte development and maturation were downregulated in AA patients, including WDR1, CASP3, MPIG6B, GATA1, CLEC1B, and ACTN1. Chemokines were upregulated in AA patients, including VCAM1, NCF1, CXCL12, CYBA, ICAM1, NCF4, and CYBB. Megakaryocyte hematopoiesis was in a state of inhibition by immune injury or inflammation in AA patients.

### Molecular Interaction of B Cell With Hematopoietic Cell in the Bone Marrow

**FIGURE 7:**
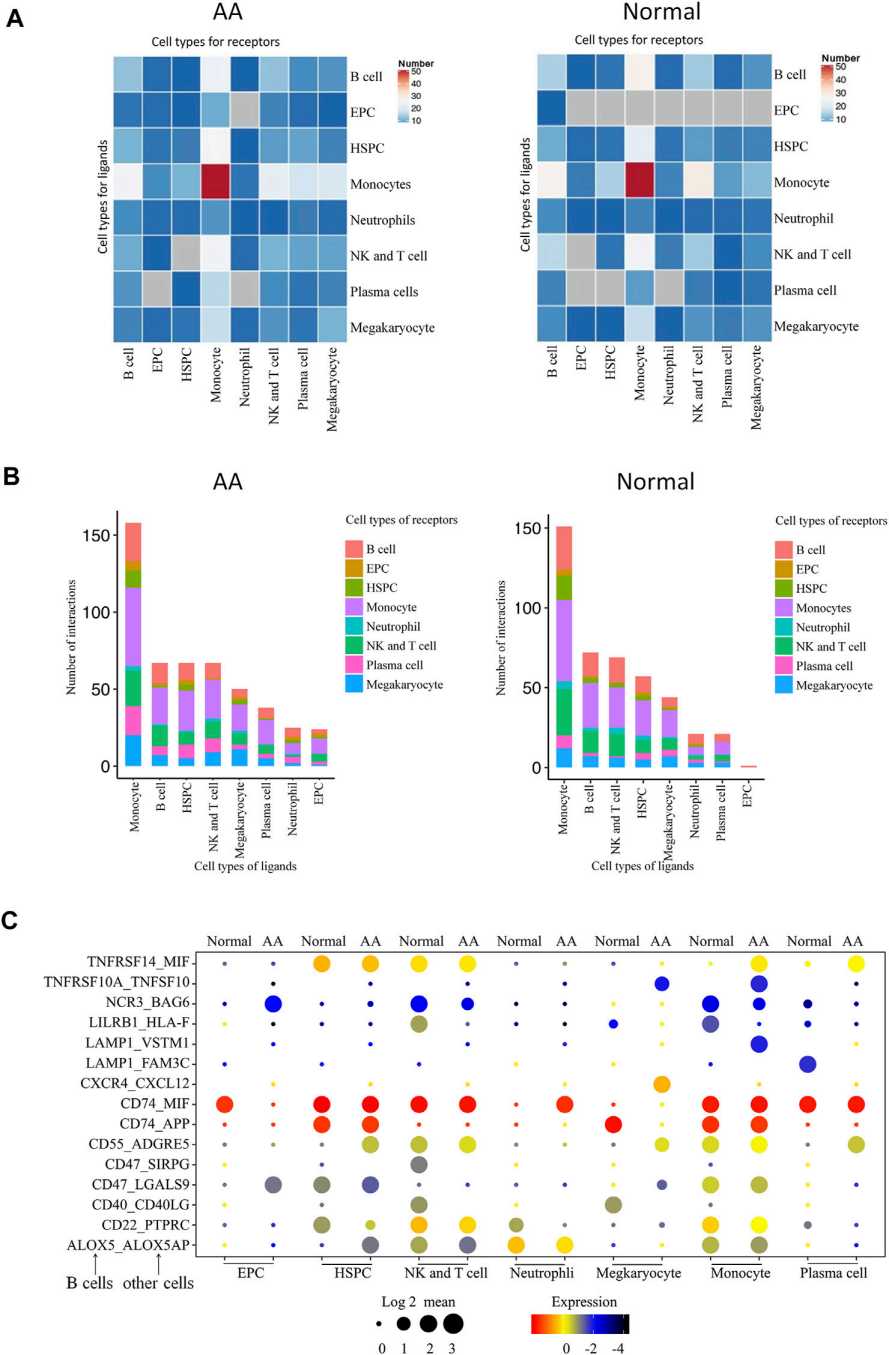
Molecular interaction of B cells with hematopoietic cells in the bone marrow. **(A)** Heatmap of the pairs of interactions between different cells. **(B)** Stacked bars of the ligand–receptor pairs interacting between cells. **(C)** Spectrum of ligand–receptor pairs between B cells (columns) and hematopoietic cells (rows). Dot sizes and colors represent logarithmic-transformed P values and mean expression of interacting molecules in corresponding cells.

A total of 496 cell type–specific ligand–receptor pairs in AA cells and 436 ligand–receptor pairs in normal cells were detected. The total number of each cell interaction was increased in patients. However, it was found that there were 67 ligand–receptor pairs in B-cell interaction with other cells in AA patients, including erythroid-like and erythroid precursor cells, HSCs, monocytes, neutrophils, NK and T cells, and platelets; there were 72 ligand–receptor pairs in healthy donors. The ligand–receptor pairs were decreased in AA patients compared with those in healthy donors, but constitution of ligand–receptor pairs was different in AA patients compared with healthy donor (Figures 7A,B). Of them, 15 ligand–receptor pairs were different comparing AA patients with healthy donors. There were four ligand–receptor pairs that were broadly activated among B cells and other cells, such as CD55_ADGRE5 that regulates the complement system, LAMP1_VSTM1 that regulates IL-17 secretion, TNFRSF10A_TNFSF10 that regulates the NKκB signal pathway, and CXCR4_CXCL12 that regulates homing of B cells (Figure 7C). There were five ligand–receptor pairs broadly inactive among B cells and other cells, such as LILRB1_HLA-F involved in inhibiting immune response, LAMP1_FAM3C involved in inflammation, CD40_CD40LG involved in anti-apoptosis signaling, CD22_PTPRC involved in the regulation of B-cell antigen receptor signaling, and CD47_SIRPG involved in preventing the maturation of immature dendritic cells and inhibiting cytokine production by mature dendritic cells. These results suggest that immune dysfunction in B cells, monocytes, neutrophils, and NK and T cells may be stimulated by B cells and accelerate bone marrow hematopoiesis failure.

## Discussion

Great advances in AA have been achieved in acknowledging its pathophysiology and performing treatment in the past decade. However, HSPC heterogeneity, BCR diversity, and VDJ gene pairing frequency in AA remain unclear.

In this study, MNCs mixed with CD34 ^+^ cells at a 4:1 ratio were classified into 17 clusters by single-cell RNA-seq. Our results are incompletely different with reported studies. A previous report classified CD34 ^+^ cells into nine clusters by single-cell RNA-seq (Zhu et al., 2021). In this study, HSPCs were found in Clusters 1, 5, 8, 10, and 13. Compared with healthy donors, upregulated genes in HSPCs were involved in signaling pathways related to lymphocyte development. A study reported that *in vitro* co-culture of patient bone marrow–derived T cells with healthy bone marrow results in significant repression of hematopoiesis (Bacigalupo et al., 1980). It was found that the top differentially expressed genes, including S100A8, TNFAIP3, and IGLL1, in HSPCs were associated with the development of lymphocytes (Du et al., 2013; Yun et al., 2018; Das et al., 2019; Lu et al., 2019). Therefore, it was speculated that HSPC tendencies to differentiate to lymphocytes may destroy hematopoiesis in patients with AA.

Abnormalities were found in monocytes and neutrophil granulocytes in patients with AA. In the KEGG pathway enrichment analysis, both cell types were associated with Th17 cell differentiation. It was reported that inflammatory factors secreted by monocytes and neutrophil granulocytes could induce the differentiation of Th17 cells (Li et al., 2018). In addition, regulators associated with the development of Th17 cells are expressed in the peripheral blood monocytes from patients with AA (Du et al., 2013). Therefore, monocytes and neutrophil granulocytes are in a state of inflammatory activation and participate in the differentiation of Th17 cells in AA patients.

Mounting evidence indicates that not only T cells but also B cells contribute to AA pathogenesis. Currently, many researchers focus on the functions and markers of T cells and B cells (Das et al., 2019; Huuhtanen et al., 2019). Studies have recently found one or more types of antibodies in AA patients that prompt B cell–mediated humoral immunity (Hirano et al., 2003; Takamatsu et al., 2009). However, BCR diversity and pairing frequency of the VDJ gene are rarely examined. It was found that three V genes (IGHV3-7, IGHV3-33, and IGLV2-11) were highly expressed in AA. It was reported that elevated expression of IGHV3-7 and IGHV3-33 is related to tissue injury in autoimmune diseases (Pramanik et al., 2011). Moreover, high pairing frequency of the V and J genes was found in virus-induced lung diseases (Wen et al., 2020). Therefore, it was speculated that B-cell participation in AA pathogenesis is related to altered V and J gene expressions.

Molecular interactions reflect the effect of B cells on the development of the hematopoietic cell of the bone marrow. It was found that the immune abnormalities of AA are related to the abnormalities of immune activation and immune negative regulation. Hence, the treatment of AA mainly focuses on the suppression of immune and immunosuppressive therapy is as the first line treatment for AA patient, now.. However, the efficacy is not satisfactory. And, a study reports that ligand–receptor pairs in AA patients treated with immunosuppression still actively interacted in HSPCs and T cells (Zhu et al., 2021). So, it was found that the changed ligand–receptor pairs suggest that immune dysfunction in B cells, monocytes, neutrophils, and NK and T cells may be stimulated by B cells, and thus accelerate bone marrow hematopoiesis failure. Both NK and T cells are derived from lymphoid progenitor cells, and they share many similarities in collotypes and function (Sun et al., 2009; Schlub et al., 2011). One must admit that there must be differences between the two. Considering the following points, there are no clear distinction: 1. Single-cell dimensionality reduction clustering often gathers cells with similar expression, and T cells and NK tend to gather together because their expression profiles are similar, especially T cells with lethal function (e.g., CD8 toxic T cells) and NK; 2. The difference between NK and T cells is often the difference of individual molecules, such as CD3d. Considering the similarity between them, they were not distinguished here.

In summary, the effects of elevated V gene expression, high pairing frequencies of the V and J genes, and change of ligand–receptor pairs on B cell functions are unknown and deserve further investigation. Data from two samples may not be enough to comprehensively analyze changes in B cell genes and BCR diversity in AA patients. Therefore, single-cell RNA-seq data will be refined in future studies by adding more samples from AA patients.

## Data Availability

The datasets presented in this study can be found in online repositories. The name of the repository and accession number can be found below: the National Center for Biotechnology Information (NCBI) Gene Expression Omnibus (GEO), https://www.ncbi.nlm.nih.gov/geo/, GSE181989.
