# Supplementary figures and images for: Single-Cell RNA-Seq of Bone Marrow Cells in Aplastic Anemia
Source: Front Genet. 2022 Jan 3;12:745483. doi: 10.3389/fgene.2021.745483 (PMC8762313; doi:10.3389/fgene.2021.745483)

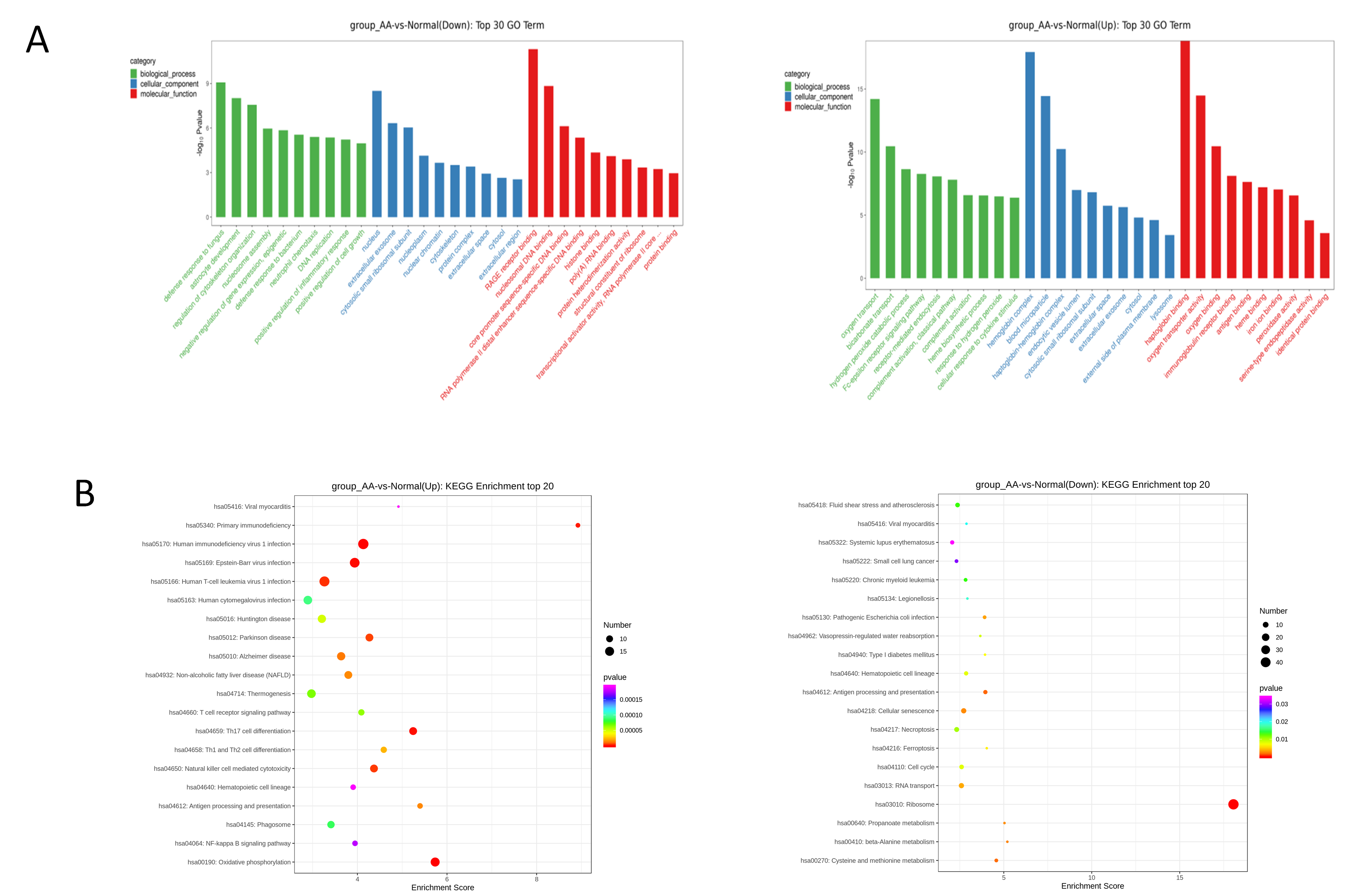

Supplement: Supplementary file 3 [file Image3.TIF]

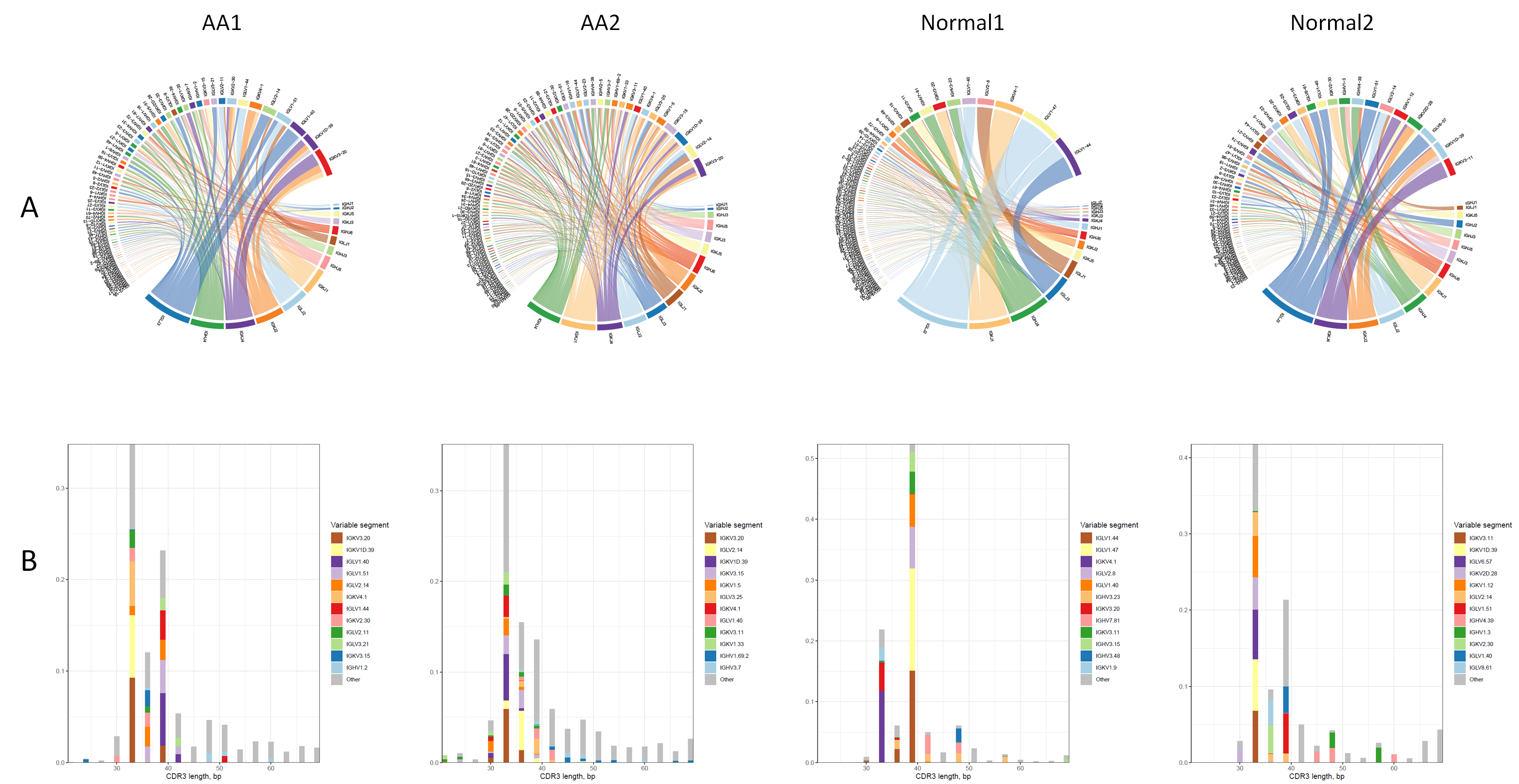

Supplement: Supplementary file 4 [file Image4.TIF]

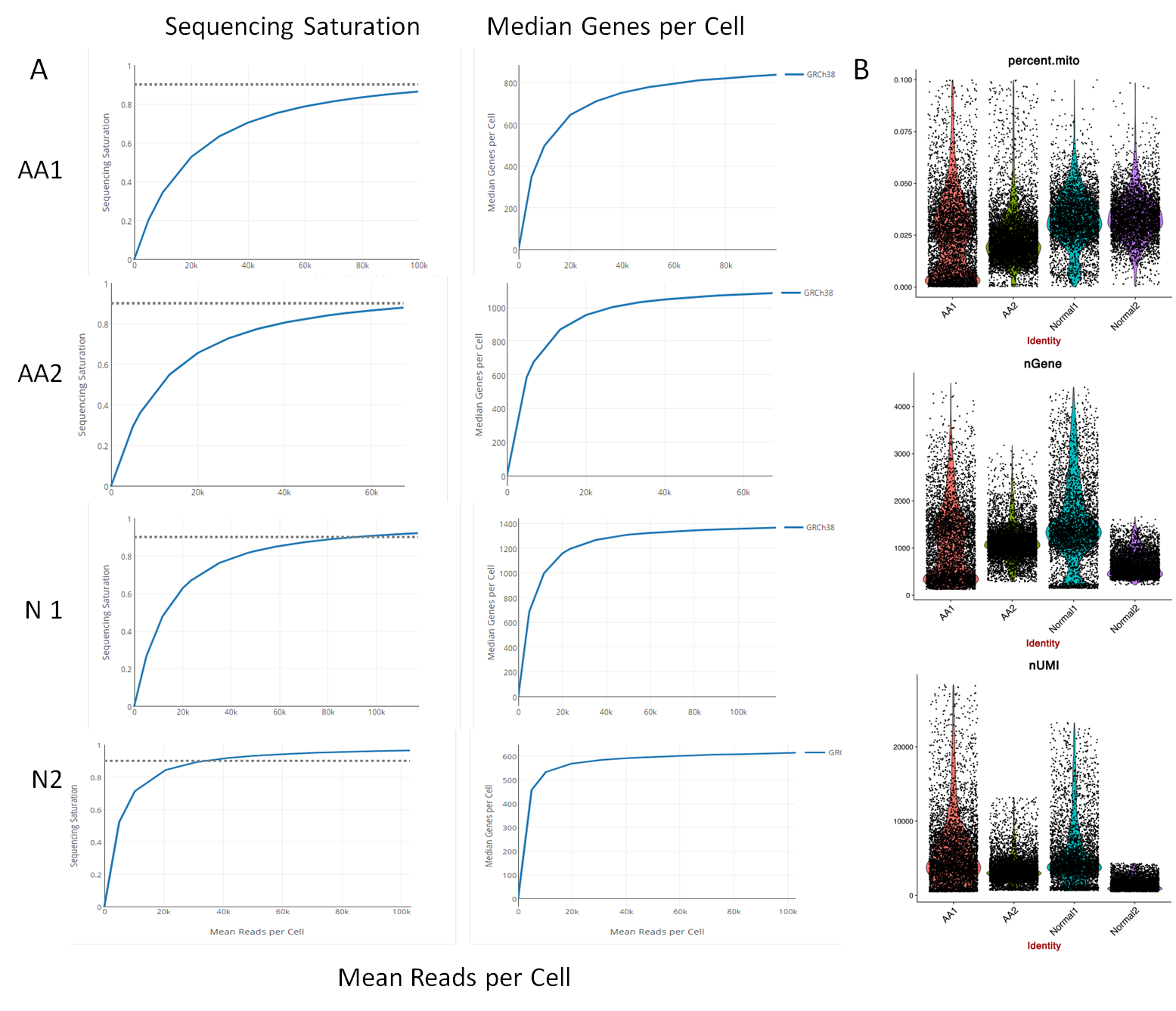

Supplement: Supplementary file 5 [file Image2.TIF]

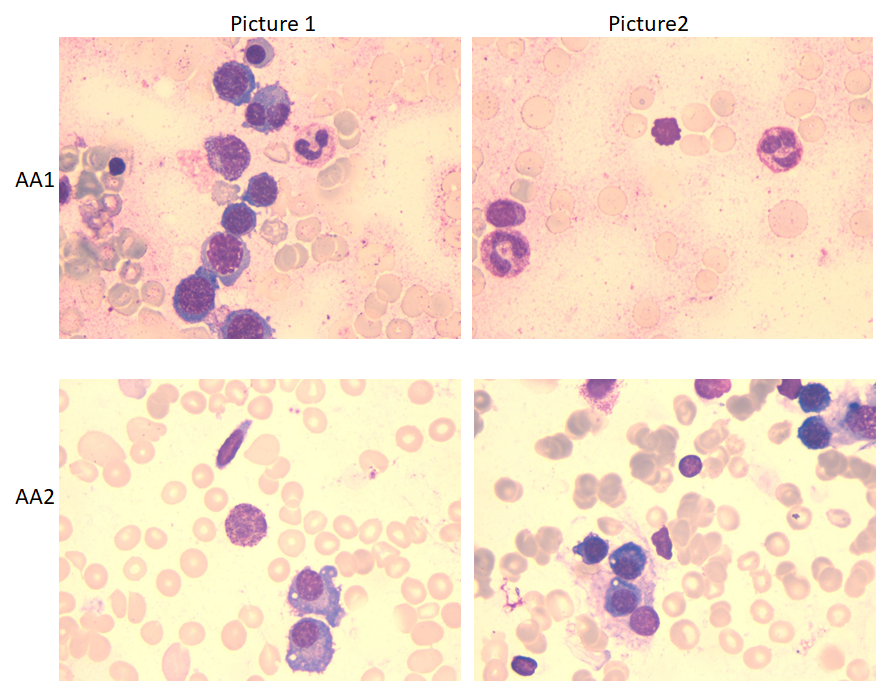

Supplement: Supplementary file 6 [file Image1.TIF]
